# Non-Adherence to Treatment Among Patients Attending a Public Primary Healthcare Setting in South Africa: Prevalence and Associated Factors

**DOI:** 10.3390/ijerph22111665

**Published:** 2025-11-03

**Authors:** Lucky Norah Katende-Kyenda

**Affiliations:** Department of Internal Medicine and Pharmacology, Faculty of Medicine and Health Sciences, School of Medicine, Walter Sisulu University, Sissons Stret, Fortgale, P Bag X 1, Mthatha 5100, EC, South Africa; nkatende-kyenda@wsu.ac.za

**Keywords:** non-adherence, primary healthcare, evidence-based nursing, primary care

## Abstract

In underdeveloped nations, treatment non-adherence continues to be a significant barrier to effective disease management. It has a major impact on patients and healthcare systems in public primary healthcare settings. Patients who do not take their medications as prescribed may be at higher risk for negative health consequences. Polypharmacy, side-effects, and drug-related problems are factors contributing to non-adherence. Additional patient-related issues include multimorbidity, lack of support, chronic-drugs, and health-literacy. The purpose of this study was to ascertain the prevalence and contributing factors of treatment non-adherence among patients presenting to a public primary healthcare setting in South Africa. Between September and October 2014, cross-sectional quantitative research using structured questionnaires was carried out with one hundred patients who were chosen using random sampling. Self-reports from patients were used to assess non-adherence to therapy. A standardized questionnaire administered by the interviewer was used to gather data, and IBM SPSS version 29 was used for analysis. Patients aged 18 years and older who were using prescribed medications were included. The characteristics of the participants were obtained using descriptive statistics, and 95% confidence intervals (CIs) are reported for Odds ratios (ORs). Associations between related factors and treatment non-adherence were obtained using the Pearson Chi-square test; a *p*-value of less than 0.05 was deemed statistically significant. Of the 100 patients interviewed, 35% were men and 65% women. The majority were in the age-range of 60–80 years with a high school level of education. Demographic characteristics associated with non-adherence to treatment were gender (*p* = 0.03) and age (*p* = 0.03). Chronic conditions, alcohol consumption, recreational drug use, use of medication reminders, waiting time to get treatment and support from healthcare providers all were statistically significant with *p*-values < 0.001, time to get to the clinic (*p* = 0.02), mode of transport (*p* = 0.01), alcohol consumption (OR 22.25 [95% CI: 8.539–57.977], *p* < 0.001) and recreational drug use (OR 8.73 [95% CI: 5.01–15.98], *p* < 0.001) were also examined. Patient medication non-adherence is a major medical problem globally. Though patient education is the key to improving compliance, use of compliance aids, proper motivation, and support are also shown to increase medication adherence.

## 1. Introduction

The simplest way to understand non-adherence is to think about how a person interacts with a particular illness or therapy in a social and environmental setting [1]. Stewart et al. (2023) [2] states that a complex interaction of socioeconomic, healthcare system, and patient variables affects non-adherence to treatment in public primary healthcare settings. Poor health literacy, ignorance about the disease and its treatment, fear of side effects, and mental health disorders like anxiety or depression are all patient-related issues, according to Martin et al. (2005) [3]. Factors associated with the healthcare system include things like inadequate communication between patients and healthcare professionals, lengthy waiting times, and restricted access to care (Al-Worafi, 2024) [4]. In their research, Burch et al. (2016) [5] found that socioeconomic variables, including unemployment, low income, and a lack of social support can also have a big influence on adherence.

Poor treatment results are linked to medication non-adherence [6]. According to the findings of several authors, including Luga and McGuire (2014) [7] and Cutler et al. (2018) [8], the burden of healthcare in terms of high resource use and extravagant costs for patients and society may increase if patients do not receive the anticipated health benefits due to medication non-adherence. Oliveira et al. (2024) [9] concluded from their study on medication adherence in adults that medication adherence is a critical component of managing chronic diseases and is critical to attaining favorable treatment results. Just 50% of patients with chronic illnesses in wealthy nations take their medications as directed, according to a 2003 World Health Organization (WHO) study [10]. The prescription of a pharmaceutical medication is one of the most prevalent interventions in industrialized health economies, where medications are necessary treatments for most chronic diseases, according to the findings of a study conducted by Bronkhorst et al. (2014) [11]. According to the WHO (2003) [12], non-adherence is still a major obstacle to getting the most out of prescription drugs, particularly for chronic illnesses.

Around half of medications prescribed for chronic diseases were not taken as directed, according to a classic study by the WHO (Sabaté, 2003) [10] and Simpson et al., 2006) [13]. According to a 2018 Organization for Economic Co-operation and Development (OECD) report [14], inadequate medication adherence is thought to be a contributing factor in almost 200,000 preventable deaths in Europe annually. The significant financial burden of non-adherence is also highlighted in this report, “Investing in medication adherence improves health outcomes and health system efficiency” which estimates that preventable hospitalizations, emergency care, and outpatient visits cost European countries approximately EUR 125 billion annually.

Significant numbers of patients, especially those with chronic diseases, do not take their prescriptions as directed, according to reports from the WHO and the OECD [15]. Similarly non-optimized medication therapy is expected to have a USD 500 billion economic effect in the United States (Burkhart and Sabaté, 2003) [16]. Adherence rates in routine clinical practice are below ideal despite these repercussions. Only 50% of patients are expected to stick with long-term therapy, according to Burkhart and Sabaté (2003) [16]. Numerous causes of non-adherence have been found since 2003 in a variety of treatment domains and nations [17,18,19]. Notably several authors disclosed that these factors may be located at the level of the patient (e.g., beliefs, cognition, comorbidities, knowledge), the treatment (e.g., side effects, dosage schedule, co-medication), or the health system (e.g., medication accessibility, communication with healthcare providers, social and IT support) [20,21,22,23].

Numerous treatments have been explored to address non-adherence, with variable degrees of success [24,25]. The great majority of these interventions concentrate on a specific component associated with non-adherence [26]. The majority of these therapies have focused on either a treatment-level barrier (such as lowering the dosage regimen) or a patient-level barrier (such as sending electronic reminders) [27]. The obstacles of the healthcare system to promoting medication adherence (MA) and healthcare professionals’ (HCPs) management of it are far less well understood. HCPs and patients highlighted that communication issues might affect MA in qualitative research conducted in the USA [28].

According to Irish HCPs, continuity of treatment was found to be a crucial requirement for MA [29]. More inter-professional involvement in patient follow-up, better patient education and IT systems, and improved care coordination were identified as major obstacles in a small study of 16 general practitioners in Finland [10]. It is unclear, however, whether these findings apply to other European nations and healthcare professionals. Only half of HCPs ask their patients about adherence, according to studies conducted throughout Europe [30], but there is still a dearth of general knowledge of the obstacles that HCPs believe stand in the way of proper MA treatment in Europe.

One of the main factors influencing the effectiveness of therapy is adherence to medicines. Non-adherence is a severe issue that affects the patient as well as the healthcare system. In their study, Jimmy and Jose (2011) [31] found that patients who do not take their medications as prescribed have significant deterioration of their illness, mortality, and higher health care expenses. Adherence may be affected by several things. In his research, Chia (2008) [32] concluded that patient, provider, and health system characteristics, together with their interconnections, may be used to alleviate adherence hurdles. Improving medicine adherence will require identifying unique obstacles for each patient and implementing appropriate strategies to get beyond them. In their regular work, medical professionals, including doctors, pharmacists, and nurses play a big part in helping patients take their medications as prescribed.

### 1.1. Conceptual Framework Correlates and Interventions

A conceptual framework can be employed in primary care research on treatment non-adherence because it offers an organized method for comprehending and examining the intricate interactions between variables that affect adherence (Stewart et al., 2023) [2]. To increase adherence, this framework may be used to pinpoint important factors, connections, and possible treatments. The best way to understand non-adherence is to look at how a person interacts with a particular illness or therapy in a social and environmental setting. The result of talent and drive is adherence. In addition to more ‘instinctive,’ intuitive, and habitual processes, motivation also includes cognitive decision-making processes.

Ability includes the mental and physical aptitude required to comply. In addition to triggers or cues to actions, which can be internal (such as experiencing symptoms) or external (such as getting a reminder), environmental and social variables also impact motivation and ability. Effective treatments are difficult to find, in part because few of them have a solid theoretical foundation, according to systematic studies of adherence interventions. Individually designed adherence assistance that addresses the unique perceptions (e.g., beliefs about sickness and treatment) and practicalities (e.g., competence and resources) impacting people’s desire and ability to adhere will be more successful.

The key variables include the following factors.

According to Baryakova et al. (2023) [33], patient-related variables include perceptions of obstacles such as cost, side effects, or absence of symptoms, as well as patients’ views, attitudes, knowledge, and comprehension of their disease and therapy.

- Treatment-related factors: According to Krousel-Wood et al. (2021) [34], they include the perceived side effects, the number of drugs, and the complexity of the treatment plan.
- Healthcare system factors: This includes the patient’s experience with the healthcare system, the quality of communication between the patient and provider, and the availability of resources and support, according to Gast et al. (2019) [20].
- Social and environmental factors include aspects such as social support, access to resources, and cultural beliefs.

Moomba et al. (2019) [35] support the statement by adding that social and environmental factors significantly influence health, encompassing both the social support systems and resources individuals have access to, as well as the cultural beliefs and norms that shape their behaviors and perceptions. These factors, alongside individual choices, collectively impact health outcomes.

According to Bolsewcz et al. (2015) [36], both industrialized and developing nations have identified several variables that contribute to poor adherence to ART. According to research by Ankomah et al. (2016) [37], financial considerations including transportation expenses and food shortages affect adherence. ART adherence may also be adversely affected by social variables, including partner support, disclosure (or lack thereof), stigma and prejudice, religion and family support, and traditional and cultural views.

### 1.2. Examples of Conceptual Frameworks

*The health belief model*: According to Holmes et al. (2024) [38], this approach focuses on how patients perceive the advantages, the severity of the sickness, and their vulnerability to it. Moreover, obstacles affect their health-related behaviors, such as treatment compliance.

*The theory of planned conduct:* According to this theory, attitudes toward conduct, subjective norms, and perceived behavioral control all have an impact on intentions to behave in a particular way. According to the perceptions and practicalities approach (PaPA): The theory of planned behavior is that behavioral intentions are influenced by attitudes toward behavior, subjective peer norms, and perceived behavioral control. To address each of these constructs, implementation interventions were chosen according to Verweij et al. (2023) [39]. This method highlights the need to address both perceptions and practicality by considering both the psychological and practical aspects of adherence.

#### Connection Between the Two Theories

Drawing from existing conceptual models and new data, Krousel-Wood et al. (2021) [34] offer a broader conceptual framework that includes time preferences, implicit attitudes, and structural determinants of health (SDOH) as new factors that could account for more variation in adherence as measured objectively and subjectively. This model offers recommendations for the planning, execution, and evaluation of treatments aimed at achieving long-term improvements in medication adherence and clinical outcomes for older men and women with hypertension.

### 1.3. Aim and Objectives

The study sought to ascertain the prevalence of treatment non-adherence and related variables among patients presenting at a South African public primary healthcare setting. This is a unique instance of improving quality and outcome in primary care with evidence-based nursing. The following objectives were used to accomplish the main aim. These objectives were to:

- Describe demographics of patients attending the clinic.
- Determine the prevalence of non-adherence to treatment.
- Identify demographic characteristics associated with non-adherence to treatment.
- Assess healthcare system factors associated with non-adherence to treatment.
- Establish patient-related factors associated with non-adherence to treatment; and
- Ascertain socio-economic factors associated to non-adherence with treatment.

## 2. Methods

### 2.1. Study Design, Area, Period

#### Selection of the Research and Study Design

This was a cross-sectional study that was observational and descriptive in nature. The design was selected because it enabled the researcher to study, appreciate and determine the factors contributing to non-adherence to treatment. The study will benefit the present and the future lives of the patients and positively impact clinical resources, including budget and therapeutic material.

### 2.2. Study Design

From 1 September to 30 October 2014, cross-sectional research was carried out in a public basic healthcare clinic in the Eastern Cape of South Africa. A standardized questionnaire was used to measure non-adherent medication to ascertain the sociodemographic characteristics and non-adherence to treatment in patients attending a public primary healthcare facility. NOTE: This study is current, but data were collected in 2014. This is appropriate for achieving the aim of this study because this study examines past events and recent data is unavailable.

### 2.3. Study Site

The study was carried out at the Mbekweni Public Primary Health Care Clinic, which is situated in Viedgesville at the mouth of Mbekweni. The clinic serves around 32,769 residents of Viedgesville, which is 15 km away from Mthatha, which is about 12 km away.

### 2.4. Study Population, Inclusion, and Exclusion Criteria

Every patient who visited the Mbekweni Primary Health Care Clinic served as the source population of the study.

*The inclusion criteria were*:

- Adult patients (18 years of age or older) who take medicine from the clinic for any disease and attend the public primary healthcare system were eligible to participate in the research.
- Both males and females.
- Participants who are open to taking part in the research; and
- People of all racial groups.

*The exclusion criteria were:*

- Individuals who declined to take part in the research.
- Individuals who were too seriously unwell to answer the interview questions; and
- Those who did not have a caretaker.

### 2.5. Sample Size Determination and Sampling Technique

#### 2.5.1. Sample Size and Calculation

A single population proportion calculation was used to calculate the sample size, assuming a 95% confidence interval, a 5% margin of error, and *p* (population) = entire group of participants of interest in this study. Therefore, the formula used to calculate sample size is Slovin’s formula [40]: *n* = N/(1 + N e^2^) where:

*n* = sample size, N = proportion of population size = 526, e = margin of error (<10%)

The confusion is cleared as follows:

n = N/(1 + N e^2^)

n = sample size

N = population = 526

e = margin of error (<10%) = 0.09

n = 526/(1 + 526) (0.09 × 0.09)

n = 526/(1 + 526) (0.0081)

n = 526/(1 + 4.2606) N and Ne^2^ are not equal: N = 526 while Ne^2^ = 4.2606

n = 526/5.2606

n = 99.988—Comment: correct number will be “n = 99.989” bringing to the nearest = 100

n = 100

Therefore, our sample size was 100.

#### 2.5.2. Data Sampling Strategy

Random sampling was the method utilized to choose research participants in this investigation to minimize selection bias, as this was a prevalence study. Targeting both male and female participants, as well as a variety of age groups, was performed to cover a wide range of characteristics that influence patient non-adherence to therapy.

#### 2.5.3. Study Variables

Medication non-adherence served as the dependent variable in the result, whereas demographics (gender, age, marital status, educational attainment, and clinical- and medication-related factors) were independent categorical variables.

### 2.6. Data Collection

#### 2.6.1. Data Collection Tool and Data Collectors

The data was collected using a structured questionnaire [41], that was designed with open-and closed-ended questions in both English and Isixhosa. The questionnaire contained variables that were assigned codes used to record data on a questionnaire. The same codes were used to capture data into Statistical Package for Social Sciences (SPSS) software for data analysis. For demographic factors, the following were coded: age range (1 = 18–39, 2 = 40–59, 3 = 60–80, 4 = >80), level of education (1 = illiterate, 2 = primary school, 3 = high school and 4 = tertiary), and gender (1 = males, and 2 = females). For both genders the following subgroups were coded: (1 = young adults, 2 = middle-aged, 3 = elderly and 4 = old age).

For patient-related factors, the following were coded: patients who have someone to remind them to take their treatment (1 = child, 2 = grandchild, 3 = mother, 4 = sibling, 5 = grandmother and 6 = sibling), socioeconomic factors (1 = alcohol usage, 2 = smoking, 3 = recreational drug usage, 4 = traditional medicine and 5 = traditional medicine and western medicine), and number of chronic diseases (1 = HPT, 2 = DM, 3 = arthritis, 4 = TB, 5 = HIV/AIDS, 6 = epilepsy, 7 = asthma, 8 = mental illness).

Patient knowledge about illness (1 = patient educated about illness, 2 = patient not educated about their illness), patient knowledge about side effects, (1 = patient educated about side-effects, 2 = patient not educated about side-effects), and non-adherence to treatment (1 = patient who take their medication daily, 2 = patient who do not take their medication daily) was collected for patients not complaining about their medication and patients complaining about their treatment. The responses given were “Yes” and “No” answers. These have been primarily used in dichotomous questions or as binary variables, providing a simple way to gather data and analyze responses. Patients’ non-adherence to treatment per illness (1 = HPT, 2 = DM, 3 = Arthritis, 4 = TB, 5 = HIV/AIDS, 6 = Epilepsy, 7 = Asthma, 8 = Mental illness) and patients’ response to treatment (1 = patients who feel better after taking treatment, 2 = people who do not feel better after taking their treatment) were also coded. The data was then collected by two research assistants, who were supervised by the lead investigator.

Non-adherence to treatment was measured using self-reporting. A questionnaire was designed with questions, and the following criteria were used to define patient non-adherence: Whether the patient missed >20% of doses, and whether the reasons for missing their doses were forgetfulness, being too busy, or concerns about side effects.

#### 2.6.2. Data Collection Techniques

Using a standardized questionnaire [41], participants were interviewed in person to achieve the goal of the study. According to the patient’s consent form, participants were told that the information and data collected from them for this study would be kept private and that their names and identification numbers would not be included in the report.

### 2.7. Data Handling and Analysis

Using a computer, data was gathered, cleaned, recorded, and examined using the SPSS statistical software. Software called SPSS 29 was used to enter the coded data that had been gathered. The characteristics of the individuals were described using descriptive statistics, and categorical variables were presented as frequencies and percentages. The relationship between independent and dependent variables was then examined using Pearson Chi-Square. Binary logistics was used to determine the association between these two factors and the odds ratio, which represents the likelihood of the binary outcome, then was ascertained by regression analysis. For every variable, the odds ratios (OR) with a 95% CI were calculated, and a *p*-value of less than 0.05 was deemed statistically significant.

#### Data Reporting

Results from the data analyzed have been presented in the form of tables, bar charts and pie charts.

### 2.8. Ethical Considerations

The study was conducted in accordance with the Declaration of Helsinki, and the protocol was approved by Walter Sisulu University, Faculty of Medicine and Health Sciences Biosafety Human Ethics Research Committee (HREC). The project identification number was 071/2014 and issued on 27 August 2014. Furthermore, permission was obtained from OR Tambo Municipality and KSD, under which the clinic operates. Finally, permission was obtained from the manager of the clinic where data was collected.

Using a participant information sheet, the research team informed the participants of the goals and advantages of the study prior to data collection. Participants were informed about the goals of the study as outlined in the proposal. The participants were then requested to sign a written consent form, which was optional, to participate in the study. The participants were told that if at any moment they felt like not continuing participating in the project, they were free to stop. The researcher and data collectors assured the participants of anonymity and confidentiality of the information as indicated in the patients’ consent form.

## 3. Results

### 3.1. Demographic Characteristics of the Study Participants

Of 100 participants interviewed, 35% were males and 65% females. The majority (45%) were in the age range of 60–80 years. Of the 100 total distribution of patients per age, it was revealed that the majority (45%) were elderly. According to the age distribution of the elderly per gender, 14 (40%) were females and 31 (47.7%) males.

In terms of education level, the majority (40%) of the participants completed high school, and of these, 26 (47.7%) were females and 14 (40.0%) males. In all demographic characteristics, the females were the majority (see Table 1).

Furthermore, the patient’s adherence to treatment was determined, and it was established that 85 (85%) were adhering to treatment while 15 (15%) were not, as shown in Table 1.

### 3.2. Patient Related Factors to Non-Adherence

Patients were interviewed about factors that contribute to non-adherence to their treatment. Responses obtained include chronic conditions, and some patients mentioned that some have people who remind them to take their treatment while others do not. Lastly, some mentioned that the number of meals they eat per day also has an influence on the non-adherence to treatment, as shown down in Table 2.

### 3.3. Factors Linked to Treatment Non-Adherence

#### 3.3.1. Demographic Characteristics Associated with Non-Adherence to Treatment

In this section, patients were asked to respond to the questions with “Yes” if they are adhering and “No” if not adhering. A result of “0” means there were no patients who responded to the questions. Otherwise, for the numbers indicated, refer to the number of patients that responded “Yes” and “No.” The demographic characteristics established to be associated with non-adherence to treatment were gender (*p* = 0.03), age (*p* = 0.03), and female and male education levels with *p* values < 0.001 as shown in Table 3.

#### 3.3.2. Factors Related to Non-Adherence to Treatment in the Healthcare System

The waiting time for patients to obtain treatment at the clinic (*p* = 0.02), the support patients got from healthcare professionals (*p* ≤ 0.001), and the method of transportation (*p* = 0.01) were all elements in the healthcare system that were linked to non-adherence to treatment. As shown in Table 4, there was no correlation between non-adherence and the total distance from home to the clinic (*p* = 0.11), or the availability of medications (*p* = 0.51).

#### 3.3.3. Patient-Related Factors Associated with Non-Adherence to Treatment

The results from the current study revealed that patient-related factors associated with non-adherence to treatment were the following: The time it takes for patients to go to the clinic (*p* = 0.02); Patients with chronic conditions, and those who have reminders to take treatment, both were statistically significant with *p*-values < 0.001. Those who remind patients to take their treatment was also statistically significant (*p* = 0.01) all demonstrated in Table 5.

#### 3.3.4. Socio-Economic Factors Associated with Non-Adherence to Treatment

The odds ratio with 95% CI lower and upper limits and Pearson (X^2^) Chi-Square (*p* value) were used to calculate the associations between socioeconomic characteristics and non-adherence. The following socio-economic factors were statistically significant with non-adherence to treatment: The odds ratio for alcohol consumption was 22.25 [95% CI: 8.539–57.977], *p* ≤ 0.001; recreational drug-use 8.727 [95% CI: 5.005–15.219], *p* ≤ 0.001; gender 0.294 [95% CI: 0.095–0.911], *p* = 0.03; and reminders 0.722 [95% CI: 0.612–0.852], *p* < 0.001. The following were not statically significant: smoking 1.88 [95% CI: 1.088–1.296], *p* = 0.33; use of traditional medicine: 0.205 [95% CI: 0.025–1.647], *p* = 0.103; and medicine availability: 1.449 [95% CI: 0.480–4.376], *p* = 0.509. This information is displayed in Table 6.

In Table 6, “Yes” and “No” answers to survey questions are categorized as nominal data. This type of data is also referred to as categorical data or binary data, particularly when there are only two options. These categories (yes/no) have no inherent order or ranking; one is not inherently better or higher than the other “Yes” and “No” answers.

It is noted in the current study that multiple testing corrections like Bonferroni or False Discovery Rate (FDR) were not applied for multiple variables comparisons. Applying Bonferroni or FDR corrections for multiple comparisons can be problematic when analyzing multiple variables, because it can lead to a loss of statistical power and an increased risk of Type II errors (false negatives). This is a limitation of this study. Furthermore, odds ratios for multiple variables were not reported, because risk estimates statistics cannot be computed. They are computed for a 2 by 2 table without empty cells (Table 6).

## 4. Discussion of Results

### 4.1. Key Findings

The current study sought to determine treatment non-adherence among patients enrolled in a public health clinic. Of the 100 participants, 35% were men and 65% were women. Of the participants, 40% completed high school, and the majority, 45%, were between the ages of sixty and 80. According to the gender breakdown, 13% of men and 26% of women had a high school diploma. There were 15% non-adherents and 85% adherents in terms of non-adherence. Health care systems, patient-related factors, socioeconomic factors, and demographic traits were all linked to non-adherence to treatment.

### 4.2. Discussion of Key Findings

According to the findings of the study, out of the 100 individuals surveyed, 65% were women and 35% were men. According to cohort research by Bonolo et al. (2013) [42], non-adherence was 1.5 times more common in women than in men, which is consistent with the findings of the current study, which showed that 35% of participants were men and 65% were women. These findings support the necessity of creating treatments in public referral centers that take gender variations into consideration. It is also underlined how crucial it is to comprehend the obstacles to obtaining and using healthcare services to attain and sustain suitable adherence levels.

Participants in this research were mostly between the ages of 60 and 80 (45%). Ghidei et al. (2013) [43], provide support for these findings, indicating that older persons are often thought to be more susceptible to pharmaceutical non-adherence, because of things like prescription complexity, side effects, expense, and cognitive loss. However, elderly people living with HIV may not be included by this generalization. According to the gender breakdown, 13% of men and 26% of women had a high school diploma.

According to the results of the current investigation, medication adherence was significantly predicted by educational attainment. One study performed by Bauer et al. (2015) claim that high levels of adherence to treatment regimens are a result of educating participants [44]. People with more education are more likely to understand the significance of drugs, which has a big effect on adherence. According to research by Sweileh et al. (2004), patients who were illiterate and could not tell the difference between their prescriptions were more likely to make mistakes, not follow their treatment plans, and had less medical understanding [45].

In terms of non-adherence, the results of this study revealed that 85% were adherent and 15% non-adherent. Walsh et al. (2019) [6] reveal that in some studies, non-adherence to treatment has been observed in the range of 85% adherence and 15% non-adherence. According to research conducted in Lesotho, 52.4% of hypertension patients missed visits, and 64.6% had at least one instance of not taking their medicine as directed, according to the Open AIDS Journal. In other cases, >85% adherence was associated with higher treatment success, while >90% adherence did not significantly differ in treatment success. In summary, while a study might show 85% adherence and 15% non-adherence, it is important to remember that non-adherence is a complex issue with varied rates and numerous contributing factors. Understanding these factors is crucial for improving treatment outcomes and patient health.

Several factors were established to be associated with non-adherence to treatment. Results from the current study established that a demographic that was significantly associated with non-adherence to treatment was gender (*p* = 0.03). These results are supported by Bonolo et al. (2013) [42], confirming that gender can be a significant factor influencing treatment adherence, with some studies indicating that women may have higher rates of non-adherence compared to men. This disparity can be linked to various factors, including differences in how men and women experience medication side effects, socioeconomic factors, and the societal pressures that women face.

Another demographic factor linked to non-adherence was age, which had a statistically significant *p*-value of 0.03. This finding is supported by a cross-sectional study conducted in Hong Kong by Kang et al. (2015) [46,47], which found that the participants were older (odds ratio [OR] 1.012, 95% CI 1.002–1.022, *p* = 0.014). Additionally, research conducted by Ghidei et al. (2013) [43] says that variables such as prescription complexity, side effects, expense, and cognitive decline are usually thought to put older persons at a higher risk for medication non-adherence.

The educational attainment of both men and women was statistically significant as a demographic component linked to treatment non-adherence (*p* < 0.001). According to the results of the current investigation, medication adherence was significantly predicted by educational attainment. These findings are consistent with research by Ampofo et al. (2020) [47], which say that persons with more education are more likely to understand the significance of drugs, which has a large effect on adherence. According to research by Sweileh et al. (2004) [45], patients who were illiterate and could not tell the difference between their medications were more likely to make mistakes, not follow their treatment plans, and had less medical expertise.

Al-Worafi, Y.M (2024) [4] supported the findings of the current study by pointing out that treatment by healthcare providers to patients, which was statistically significant with a *p*-value < 0.001, is a healthcare system factor that is associated with non-adherence to treatment. Al-Worafi Y.M. (2024) [4] further support this study by emphasizing that healthcare-system-related factors encompass issues like limited access to care, long waiting times, and poor communication between healthcare providers and patients. A comprehensive understanding of HCPs’ perceived barriers to appropriate MA management in Europe is still lacking, even though surveys conducted throughout the continent have shown that only half of them ask their patients about adherence (Hafez et al., 2024) [30].

Alinaitwe et al. (2025) [48,49] say that approximately two out of three (68.7%) of the participants reported a high level of perceived family support, with family members reminding patients to take their medication (65.3%) and providing material support (55.8%) as the most common forms of family support for patients who are likely to forget to take their treatment. The statistically significant value of *p* = 0.01 in the current study aligns with studies that show that individuals who use reminders have significantly higher adherence rates and increased odds of adherence compared to those who do not use reminders.

Patient-related factors associated with non-adherence to treatment include the time it takes for patients to go to the clinic. Results in this study were statistically significant (*p* = 0.02). Results in this study agree with authors Nhlongolwane and Shonisani (2023) [49], who deduce that a significant association exists between the time it takes patients to access healthcare facilities and their adherence to treatment, particularly medication adherence. Long travel times can create logistical challenges for patients, especially those with demanding work or family responsibilities. The time spent traveling can make it difficult to fit in necessary appointments and medication refills, leading to missed doses and potential health complications. Economic factors like transportation costs and the lack of food interfere with treatment adherence, according to a study by Ankomah et al. (2016) [37] supporting the findings of this study, which found a significant association (*p* = 0.01) between modes of transportation from home to the clinic.

The current study performed an analysis of chronic conditions that patients are suffering from, which were mainly chronic conditions such as hypertension, diabetes mellitis, etc. There was an association between the chronic illnesses patients suffer from, like HPT, with statistically significant *p* values of ≤0.001. These results are supported by the WHO report in 2003 that identifies that only 50% of patients with chronic diseases in developed countries take their medication as prescribed. Furthermore, another WHO report (WHO, 2003) [12] says that non-adherence remains a significant barrier to achieving optimum outcomes from appropriately prescribed medicines, especially in long-term conditions experienced by patients in old age.

Other factors associated with non-adherence to treatment are socioeconomic factors like alcohol consumption, smoking cigarettes, and using reactional drugs. In this study, patients analyzed for associations revealed significant values in terms of odd ratios (OR), with 95% confidence intervals with upper and lower limits. Results obtained were statistically significant, resulting in an odds ratio of 8.53 (95% CI: 8.539–57.997), *p* ≤ 0.001. These results are supported by a study by Velloza et al. (2020) [50], which investigates and confirms that alcohol consumption, particularly at harmful or hazardous levels, is significantly associated with non-adherence to treatment, particularly for antiretroviral therapy (ART).

One of the studies performed by Kalichman et al. (2022) [51] stated that alcohol use, even moderate amounts, is linked to a decrease in medication adherence, and hazardous drinking is associated with a fourfold increase in the likelihood of non-adherence. In this study, the odds ratio of 22.25 (95% CI: 8.539–57.977), *p* < 0.001, obtained for alcohol consumption was high. This means that individuals who consume alcohol were significantly more likely to be non-adherent to their prescribed treatment compared to those who do not consume alcohol. Furthermore, the data was skewed due to the small sample size used in this study. The odds ratio for those who use recreational drugs was 8.727 (95% CI: 5.005–15.219), with a *p*-value of less than 0.001. This means that an (OR) of 8.727 with a 95% confidence interval (CI) of 5.005–15.219 indicates a strong positive association between the variables being studied. Specifically, it means that the odds of the outcome occurring are estimated to be 8.727 times higher for individuals in the exposed group compared to the unexposed group. In our study, the prevalence of adherence issues was 44.8% among non-users and 64.1% among recreational drug users. A statistically significant correlation between recreational drug use and lower adherence rates was found by Velloza et al. (2020) [50], based on the findings of the multivariate logistic regression.

The number of meals patients eat was another socioeconomic element linked to treatment non-adherence. The study found statistically significant results (*p* = 0.002). This study is supported by the findings of a study by Kalichman et al. (2011) [51], which showed that almost half of non-adherent participants reported recent hunger and that 45% of participants were less than 85% adherent to their prescriptions. Food insufficiency was therefore linked to non-adherence.

### 4.3. Strengths and Limitations

A study on non-adherence in South Africa offers valuable insights into healthcare practices and patient behavior, but is also subject to limitations.

#### 4.3.1. Strengths

*Real-world setting:* Studying a public primary healthcare setting provided a realistic view of the challenges and opportunities within the South African healthcare system according to the provided search results.

*Actionable findings:* The study identified factors contributing to non-adherence, which can inform strategies for improving adherence and treatment outcomes according to the provided search results.

*Focus on a specific population*: The study focused on a specific population, which allows for a more targeted approach to interventions according to the provided search results.

*Contribution to knowledge:* The study added to the understanding of non-adherence patterns and associated factors, which can be used to improve public health policies and interventions according to the provided search results.

#### 4.3.2. Limitations

*A sample size of 100 in this study:* This was relatively small to support strong statistical conclusions, particularly when reporting odds ratios with narrow confidence intervals.

*Self-reports used to determine adherence in this study:* This was a limitation because it introduces recall bias and social desirability bias.

*Potential for bias:* The current study might have been biased by factors such as patient selection, data collection methods, and interpretation of results, according to the provided search results.

*Reliance on self-reported data*: Self-reported adherence might not have been accurate in reflecting actual medication-taking behavior, according to the provided search results.

*Challenges in generalization*: Findings from a single setting may not be generalizable to other healthcare settings or populations according to the provided search results.

*Potential for confounding variables*: According to the search results, adherence may be influenced by several factors, including socioeconomic position, cultural attitudes, and health literacy, which can also confuse the link between the identified factors and adherence.

### 4.4. Implications and Recommendations

#### Implications

*Poor health outcomes:* Non-adherence can lead to treatment failure, increased disease severity, and higher rates of morbidity and mortality.

*Increased healthcare costs*: Non-adherence can result in more frequent hospitalizations, extended length of stay, and the need for more expensive treatments.

*Transmission of infectious diseases*: In the context of HIV/AIDS, non-adherence can contribute to viral resistance and continued transmission of the disease.

*Reduced quality of life:* Non-adherence can negatively impact patients’ quality of life by leading to increased symptoms, anxiety, and a sense of loss of control.4.4.2. Recommendations from This Study

*Tailored interventions:* Create and carry out therapies that are suited to the unique requirements and conditions of various patient groups.

*Improve patient education*: Clearly and succinctly explain to patients their prescriptions, treatment plans, and the value of adherence.

*Address psychosocial factors*: Identify and address psychosocial barriers to adherence, such as stress, anxiety, depression, and the lack of social support.

*Utilize technology*: Utilize technology to support adherence, such as reminder apps, pillboxes, and telehealth.

*Strengthen healthcare provider–patient relationships*: Foster strong and trusting relationships between healthcare providers and patients.

*Promote adherence at the community level:* Engage community organizations and leaders to promote adherence and address social determinants of health.

*Conduct ongoing research:* Conduct ongoing research to identify effective strategies for improving adherence and address emerging challenges.

## 5. Conclusions

In order to highlight the impact of non-adherence on health outcomes and to guide the development of strategies to improve adherence, a study on medication non-adherence can provide important insights into factors influencing adherence and possible interventions. For instance, studies may identify systemic, social, and psychological factors that contribute to non-adherence, such as complex treatment regimens, lack of knowledge, or financial constraints.

According to The Open AIDS Journal, non-adherence to HIV treatment with ARTs can result in treatment failure, drug resistance, and an increased risk of AIDS-related deaths. Similarly, non-adherence to cancer treatments (e.g., endocrine therapy) can be associated with an increase in all-cause mortality; and non-adherence to medications for chronic diseases such as diabetes and cardiovascular disease (CVD) can also have a negative impact on health outcomes.

Determining the sociodemographic elements that influence non-adherence, such as age, gender, and educational attainment, is crucial. Patient-related elements, such as the length of time it takes for patients to visit the clinic and the quality of care or assistance they receive from the medical staff, also play a role. Socioeconomic elements, such as teaching patients about the risks associated with recreational drug use and alcohol use, are important.

## Figures and Tables

**Table 1:** Socio-demographic of the study participants (*n* = 100).

Variable	Frequency (*n*)	Percentage (%)
**Gender**		
Male	35	35.0
Female	65	65.0
**Total**	**100**	**100.0**
**Age range**		
18–39	15	15.0
40–59	37	37.0
60–80	45	45.0
>80	3	3.0
**Total**	**100**	**100.0**
**Total distribution per age category**		
**Youth**	15	15.0
Middle age	37	37.0
Elderly	45	45.0
Old age	3	3.0
**Total**	**100**	**100.0**
**Age distribution of participants per gender (male)**		
Youth	8	22.9
Middle Age	11	31.4
Elderly	14	40.0
Old Age	2	5.7
**Total**	**35**	**100.0**
**Age distribution of participants per gender (female)**		
Youth	7	10.8
Middle age	26	40.0
Elderly	31	47.7
Old age	1	1.5
**Total**	**65**	**100.0**
**Education level**		
Illiterate	28	28.0
Primary	30	30.0
High School	40	40.0
Tertiary	2	2.0
**Total**	**100**	**100.0**
**Distribution of education level per gender (females)**		
**Female education level**		
Illiterate	17	26.2
Primary	21	32.3
High school	26	40.0
Tertiary	1	1.5
**Total**	**65**	**100.0**
**Male education level**		
Illiterate	11	31.4
Primary	9	25.7
High School	14	40.0
Tertiary	1	2.9
**Total**	**35**	**100.0**
**Patients’ adherence to treatment**		
**Adherence**	85	85
**Non-adherence**	15	15
**Total**	**100**	**100.0**

**Table 2:** Number of patients and contributing factors to non-adherence.

Number of Patients with Chronic Conditions Who Were Non-Adherent	n	Number of Patients Who Stated Not Having the Mentioned People to Remind Them to Take Their Treatment and thus Are Not Adhering to Treatment	n	Number of Patients Who Responded That Due to Lack of the Number of Meals Are Not Adhering to Their Treatment	n
Condition		Reminder		Number of Meals	
Hypertension	8	Child	17	1	2
Diabetes	2	Spouse	17	2	21
Tuberculosis	2	Mother	4	3	63
HIV/AIDS	2	Sibling	2	4	11
Mental illness	1	Grandmother	1	5	3

**Table 3:** Demographic characteristics associated with non-adherence to treatment.

Variable Categories	Adhering/Non-Adherence			Pearson Chi-Square Tests	
	Yes	No	Total	X^2^	*p* Value
*Gender*				4.848	***0.03***
Female	6	59	65		
Male	9	26	35		
Total	15	85	100		
*Age*				8.925	***0.03***
16–39	4	11	15		
40–59	4	33	37		
60–80	5	40	45		
>80	2	1	3		
Total	15	85	100		
*Education level*				2.110	0.55
Illiterate	6	22	28		
Primary	5	25	30		
High school	4	36	40		
Tertiary	0	2	2		
Total	15	85	100		
*Female education level*				*55.952*	*<* **0.001**
Illiterate	15	2	17		
Primary	0	21	21		
High school	0	26	26		
Tertiary	0	1	1		
Total	15	50	65		
*Male education level*				20.880	**<0.001**
Illiterate	11	0	11		
Primary	0	9	9		
High school	4	10	14		
Tertiary	0	1	1		
Total	15	20	35		

**Table 4:** Healthcare system factors associated with nonadherence to treatment.

Variable Categories	Adherence/Non-Adherence			Pearson Chi-Square Tests X^2^	*p* Value
	Yes	No	Total		
Waiting time to get treatment				14.840	**0.02**
0–30 min	15	13	28		
31–60 min	0	69	69		
91–120 min	0	3	3		
121–180 min	15	85	100		
181–210 min					
200–240 min	9	64	73		
241–300 min	2	19	21		
Total	3	2	5		
Support from healthcare providers	0	0	0	45.378	**<0.001**
Acceptable	14	85	100		
Good					
Bad	5	53	58		
Total	7	22	29		
Mode of transport	3	10	13	9.227	**0.01**
Taxi	15	95	100		
Walking					
Own Car	7	32	39		
Horse	8	53	61		
Total	15	85	100		
Distance from home to the clinic	15	13	28	4.416	0.11
>5 km	0	69	69		
<5 km	0	3	3		
Walking distance	15	85	100		
Total					
Medicine availability	9	64	73	0.436	0.51
Not always available	2	19	21		
Always available	3	2	5		
Total	0	0	0		

**Table 5:** Patient-related factors associated with non-adherence to treatment.

Variable Categories	Adherence/Non-Adherence			X^2^	*p* Value
	Yes	No	Total		
Time it takes for patients to go to the clinic				10.083	**0.02**
0–15 min	4	27	31		
16–30 min	2	36	38		
31–45 min	4	5	9		
46–60 min	5	17	22		
Total	15	85	100		
Patients with chronic conditions				40.595	**<0.001**
Hypertension	5	22	27		
Diabetes mellitis	0	25	25		
Arthritis	0	10	10		
Tuberculosis	0	10	10		
HIV/AIDS	0	10	10		
Epilepsy	3	6	9		
Asthma	6	2	8		
Mental illness	1	0	1		
Total	15	85	100		
Patients with reminders to take their treatment				15.033	**<0.001**
Yes	15	39	54		
No	0	46	46		
Total	15	85	100		
Who reminds patients to take their treatment				14.964	**0.01**
Child	2	15	17		
Grand child	3	0	3		
Mother	1	3	4		
Sibling	2	0	2		
Grandmother	0	1	1		
Spouse	5	12	17		
Total	13	31	44		

**Table 6:** Association between socio-economic factors and non-adherence to treatment.

Categorical Variables		Adherence/Non-Adherence			X^2^	*p* Value	OR	95% CI Lower Higher Bounds
		Yes	No	Total				
Alcohol consumption					70.037	**<0.001**	22.25	8.539–57.977
Yes		11	–	11				
No		4	85	89				
Total		15	85	100				
Smoking					0.929	0.33	1.188	1.088–1.296
Yes		0	5	5				
No		15	80	95				
Total		15	85	100				
Recreational drugs use					23.611	**<0.001**	8.727	5.005–15.219
Yes		4	0	4				
No		11	85	96				
Total		15	85	100				
Traditional medicine					2.658	0.103	0.205	0.025–1.647
Yes		1	22	23				
No		14	63	77				
Total		15	85	100				
Gender					4.846	0.03	0.294	0.095–0.911
Female		6	59	65				
Male		9	26	35				
Total		15	85	100				
Patients with reminders					15.033	**<0.001**	0.722	0.612–0.852
Yes		15	39	54				
No		0	46	46				
Total		15	85	100				
Medicine availability					0.436	0.51	1.449	0.480–4.376
Not always available		7	32	39				
Always available		8	53	61				
Total		15	85	100				
Meals taken per day					17.291	**0.002**	-	-
One meal		2	0	2				
Two meals		3	18	21				
Three meals		6	57	63				
Four meals		4	7	11				
Five meals		0	3	3				
Total		15	85	100				

NOTE: Variables indicated as 5 means the number of patients that responded as adherents and non-adherents. Case in question, regarding “Traditional medicine,” there was only 1 person that responded “Yes” to adherence.

## Data Availability

Because they contain information that might jeopardize research participants’ privacy, the data supporting the conclusions are not publicly available.
